# Decreased Levels of Thioredoxin *o1* Influences Stomatal Development and Aperture but Not Photosynthesis under Non-Stress and Saline Conditions

**DOI:** 10.3390/ijms22031063

**Published:** 2021-01-21

**Authors:** Antonio Sánchez-Guerrero, Miquel Nadal, Igor Florez-Sarasa, Miquel Ribas-Carbó, José G. Vallarino, Sabrina De Brasi-Velasco, Alisdair R. Fernie, Jaume Flexas, Ana Jiménez, Francisca Sevilla

**Affiliations:** 1 Department of Stress Biology and Plant Pathology, CEBAS-CSIC, 30100 Murcia, Spain; asguerrero989@gmail.com (A.S.-G.); sdebrasi@cebas.csic.es (S.D.B.-V.); ajimenez@cebas.csic.es (A.J.); 2 Grup de Recerca en Biologia de les Plantes en Condicions Mediterranies, Universitat de les Illes Balears, 07122 Palma de Mallorca, Spain; m.n.nadal92@gmail.com (M.N.); mribas@uib.cat (M.R.-C.); jaume.flexas@uib.es (J.F.); 3 Center for Research in Agricultural Genomics (CRAG)- CSIC-IRTA-UAB-UB, Campus University of Barcelona, 08193 Barcelona, Spain; igor.florez@cragenomica.es; 4 Max Planck Institute of Molecular Plant Physiology, 14476 Potsdam-Golm, Germany; Vallarino@mpimp-golm.mpg.de (J.G.V.); Fernie@mpimp-golm.mpg.de (A.R.F.)

**Keywords:** antioxidants, oxidative stress, photosynthesis, salinity, stomata, thioredoxin *o*1

## Abstract

Salinity has a negative impact on plant growth, with photosynthesis being downregulated partially due to osmotic effect and enhanced cellular oxidation. Redox signaling contributes to the plant response playing thioredoxins (TRXs) a central role. In this work we explore the potential contribution of Arabidopsis TRX*o*1 to the photosynthetic response under salinity analyzing Arabidopsis wild-type (WT) and two *Attrxo1* mutant lines in their growth under short photoperiod and higher light intensity than previous reported works. Stomatal development and apertures and the antioxidant, hormonal and metabolic acclimation are also analyzed. In control conditions mutant plants displayed less and larger developed stomata and higher pore size which could underlie their higher stomatal conductance, without being affected in other photosynthetic parameters. Under salinity, all genotypes displayed a general decrease in photosynthesis and the oxidative status in the *Attrxo1* mutant lines was altered, with higher levels of H_2_O_2_ and NO but also higher ascorbate/glutathione (ASC/GSH) redox states than WT plants. Finally, sugar changes and increases in abscisic acid (ABA) and NO may be involved in the observed higher stomatal response of the TRX*o*1-altered plants. Therefore, the lack of *AtTRXo1* affected stomata development and opening and the mutants modulate their antioxidant, metabolic and hormonal responses to optimize their adaptation to salinity.

## 1. Introduction

Soil and water salinity are major threat to global food security since it is one of the most decisive environmental stresses affecting plant growth and productivity. Salinity causes serious physiological, biochemical and genetic changes that alter several cellular processes in plants which require the activation of repair and protective mechanisms to allow their survival. Understanding the mechanisms of plant salinity tolerance is thus a critical step in the development of stress tolerant crops [1,2]. Salinity and water stress negatively affect plant carbon balance to an extent that depends on the duration and the severity of the stress imposed. The effects on photosynthesis are most usually related to the stomatal and mesophyll CO_2_ diffusion limitations, therefore limiting photosynthetic carbon assimilation, although under severe stress conditions, photochemical and/or biochemical impairment of photosynthesis can also occur [3,4]. Besides, the response to salinity and other environmental stresses is frequently associated with an increase of reactive oxygen and nitrogen species (ROS, RNS) which can alter both, metabolite levels and cysteine thiols of proteins. These redox changes are an important and integral part in plant signaling elicited by specific stresses as well as in response to a change in energy balance [5,6]. It is well established that the cellular redox homeostasis is disturbed under salinity stress [1,7] and mitochondria as well as chloroplasts, peroxisomes and apoplast are key organelle/compartments in the ROS generation induced as a response to salinity [8]. Indeed, plant mitochondria play an important role in the salinity response by modifying the tricarboxylic acid (TCA) cycle, the electron transport pathways activity and the transport of metabolites across the inner mitochondrial membranes [9,10]. Moreover, several enzymes involved in the TCA cycle are prone to redox regulation [11], thus it is expected that mitochondrial redox regulatory proteins can play an important role in the salinity response. The main systems based on the modulation of thiol reduction are related to proteins such as thioredoxins (TRXs) and glutaredoxins (GRXs) [12,13]. Thioredoxins are ubiquous small oxido- reductase proteins (around 14 KDa) involved in the regulation of redox status of specific so-called target proteins. TRXs are present in almost all subcellular compartments [7,13,14,15]. Our understanding of the chloroplast and cytosol TRX systems has grown significantly in comparison to that of the mitochondrial ones. In chloroplasts, TRXs isoforms act as redox regulatory factors with central roles in multiple processes including plastid biogenesis, acclimation of chloroplasts metabolism to rapid changes of light intensities, reductive activation of Calvin–Benson cycle enzymes and salinity tolerance [16,17,18,19]. To date, the best-known mitochondrial TRXs are of the *o*-type although a poplar Trx*h*2 was also described in the organelle [20]. In Arabidopsis, the *TRXo1* gene has been confirmed to encode a mitochondrial TRX, whereas the localization of its paralog TRX*o*2 has not been determined yet [13,21,22]. However, in *Pisum sativum* the mitochondrial TRX*o*1 protein has been also confirmed to be localized in the nucleus [22,23]. Through a biochemical approach, PsTRX*o*1 was reported to be involved in the response mechanism to salinity stress in pea, together with its target proteins such as mitochondrial AOX, PRXII F and other mitochondrial antioxidant enzymes [22,24]. Further, genetically modified TRX*o*1 plants (*Attrxo1* T-DNA mutants) have been used to unravel the regulatory mechanisms by which the mitochondrial TRX system regulates TCA cycle enzymes, photorespiration, and mitochondrial electron transport pathways in vivo [10,11,25,26,27]. In addition, mitochondrial TRX*o*1 has been reported to be required for the proper functioning of the antioxidant metabolism under salinity and drought [10,28] and positively influence the whole plant response to salinity stress [29]. However, the potential contribution of this mitochondrial TRX*o*1 on key processes occurring in other cellular organelles such as chloroplasts, specifically in the photosynthetic response under adverse situations such as salinity remains unknown. Reduced CO_2_ assimilation results in increased ROS production under stress [4] and also, altered ROS/antioxidant responses may directly affect stomatal and mesophyll conductance to CO_2_ thus altering photosynthesis [30]. We therefore hypothesize that plants with genetically altered levels of TRXs may display effects on photosynthesis and its components since TRXs are linked to the oxidative/antioxidant and redox reactions of different cellular compartments [12,23,31,32,33]. In this study, carried out in different growth conditions as previously reported for these plants, we compare the photosynthetic gas-exchange, stomatal and mesophyll conductance as well as the oxidative, antioxidant and metabolic acclimation to salinity stress (100 mM NaCl) of two T-DNA insertion *Attrxo1* lines previously described as knock-out (KO) [28,33] and WT plants. Moreover, we also studied stomatal density and maturity as well as pore size of these genotypes in both, control and saline conditions. Collectively our results show that *Attrxo1* mutant plants displayed a non-altered photosynthetic activity after salinity stress in parallel to some adaptative changes in their metabolic and antioxidant response. In addition, evidence is provided that the decreased level of TRX*o*1 affects the stomatal development and opening. 

## 2. Results

### 2.1. Growth Parameters

**Figure 1:**
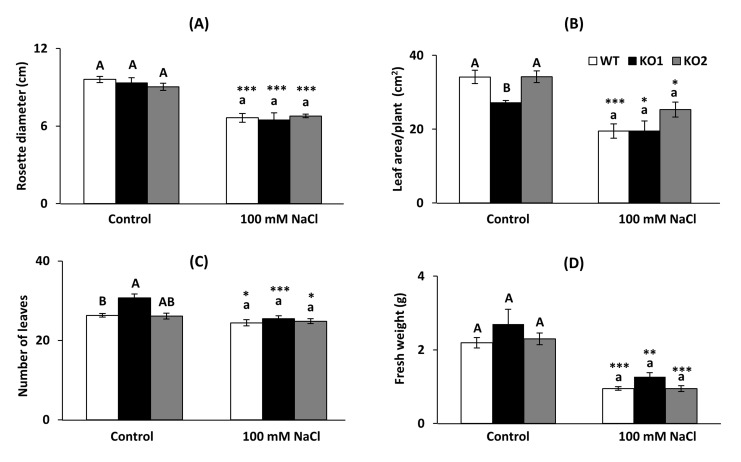
Phenotypic characterization of growth parameters. (**A**) Rosette diameter, (**B**) total leaf area, (**C**) number of leaves and (**D**) fresh weight of the aerial part of *A. thaliana* plants. Wild type plants (WT) and two *Attrxo1* mutant lines (KO1 and KO2) were grown in the absence (Control) and presence of 100 mM NaCl. Data are the mean ± SE of at least eight independent experiments. Different letters indicate significant differences (*p* < 0.05) among genotypes in each condition according to the Tukey’s test, and the asterisks indicate significant differences of each genotype in salinity compared to the control condition using the *t*-Student’s test (*, **, *** at *p* < 0.05, *p* < 0.01 and *p* < 0.001, respectively).

Visual inspection did not allow us to establish a clear phenotype caused by the decreased levels of *AtTRXo1* under non saline (control) or saline growth conditions (Figure S1), although detailed measurement of growth parameters such as rosette diameter, total foliar area per plant, number of leaves and fresh weight of the aerial part revealed some significant differences (Figure 1). Under control conditions, rosette diameter was similar in all genotypes (Figure 1A), the total leaf area was lower in the KO1 mutant than in WT and KO2 plants (Figure 1B) and KO1 line displayed a higher number of leaves (Figure 1C). Fresh weight of the aerial part was similar in all genotypes (Figure 1D). Under salinity, all genotypes displayed a decrease in the measured parameters and no significant differences were found among them in any of the parameters. 

### 2.2. Stomatal Characterization and Chlorophyll Content

**Figure 2:**
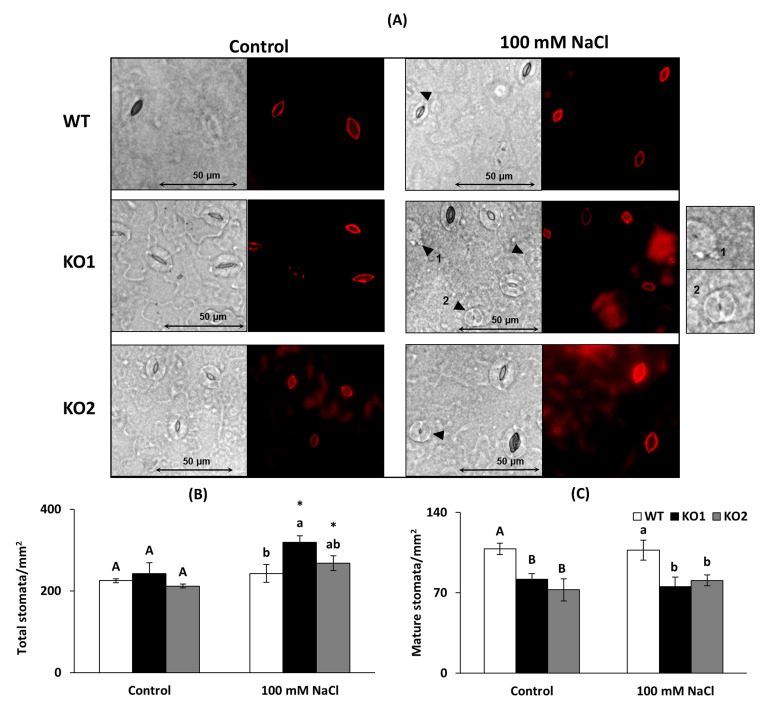
Stomatal characterization. (**A**) Foliar epidermis of *A. thaliana* wild type (WT) and two KO *Attrxo1* mutant lines grown in the absence (Control) and presence of 100 mM of NaCl. Density of (**B**) total (immature and mature) and (**C**) mature stomata. Pictures were captured with a clear field microscope and a 40× objective. Data are the mean ± SE of three independent experiments (*n* > 2000 stomata per genotype and treatment). Different letters indicate significant differences (*p* < 0.05) among genotypes in each condition according to the Tukey’s test, and the asterisks indicate significant differences of each genotype in salinity compared to the control condition using the *t*-Student’s test (*p* < 0.05). Arrows point some immature stomata and two of them (1 and 2) are shown in detail.

Figure 2A shows a representative example of the abaxial leaf epidermis from the three genotypes grown in the absence (Control) and presence of 100 mM NaCl and stained with rhodamine for a better pore visualization and measurement. We measured total stomata as immature and mature structures, considering immature all the intermediate structures through the stomatal lineage while mature stomata are the ones presenting fully developed guard cells and pore [34,35]. Under control conditions, mutant plants displayed a similar number of total stomata (Figure 2B), but less mature ones compared to WT plants (24% and 32% of decrease in KO1 and KO2, respectively, Figure 2C). Under saline conditions, KO plants displayed a higher number of total stomata (only significant in KO1 with 31% of increase) and less mature ones (29% and 24% of decrease in KO1 and KO2, respectively). Some immature stomata are pointed with arrows in the photographs. Salinity increased the number of total stomata in the KO lines but not in WT plants and this condition did not provoke a significant change in the number of mature stomata in any genotype.

**Figure 3:**
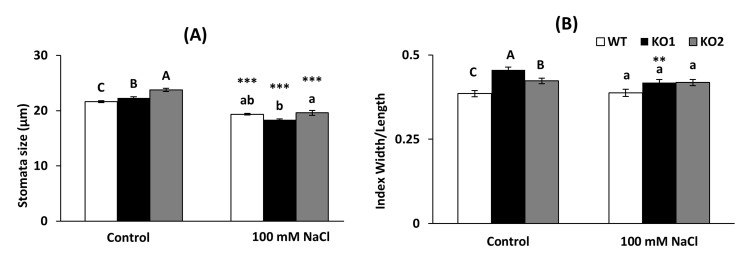
(**A**) Stomatal size and (**B**) opening index measured on the abaxial epidermis of the leaves in *A. thaliana* wild type (WT) and two KO *Attrxo1* mutant lines grown in the absence (Control) and presence of 100 mM of NaCl. The aperture index represents the measurement of the width/length of the stomatal pore. Data are the mean ± SE of three independent experiments (*n* > 500 stomata per genotype and treatment). Different letters indicate significant differences (*p* < 0.05) among genotypes in each condition according to the Tukey’s test, and the asterisks indicate significant differences of each genotype in salinity compared to the control condition using the *t*-Student’s test (**, *** at *p* < 0.01 and *p* < 0.001, respectively).

The size of the stomata was higher in both KO leaves than in WT under control conditions (Figure 3A) while not significant differences were found under salinity, a condition that decreased the size of stomata in all genotypes. As for the stomatal opening, the KO mutants displayed higher index of opening under control conditions than the WT while the opening was similar in all genotypes under salinity conditions (Figure 3B). Only the KO1 genotype presented a significant reduction in stomatal opening under salinity compared to control conditions.

**Figure 4:**
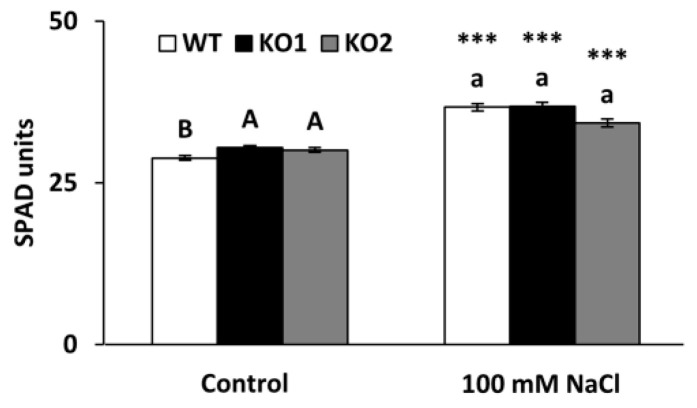
Total chlorophyll content in *A. thaliana* wild type (WT) and two KO *Attrxo1* mutant lines grown in the absence (Control) and presence of 100 mM of NaCl. Data in SPAD units are mean ± standard error of at least eight independent experiments. Different letters indicate significant differences (*p* < 0.05) among genotypes in each condition according to the Tukey’s test, and the asterisks indicate significant differences of each genotype in salinity compared to the control condition using the *t*-Student’s test (*p* < 0.001).

Total chlorophyll was estimated by SPAD measurements which revealed a slight although significant increase (5%) in the KO plants under control conditions compared to WT plants. Salinity led to an increase in chlorophyll content in all genotypes, but no significant differences were observed among them under this condition (Figure 4).

### 2.3. Oxidative Markers 

**Figure 5:**
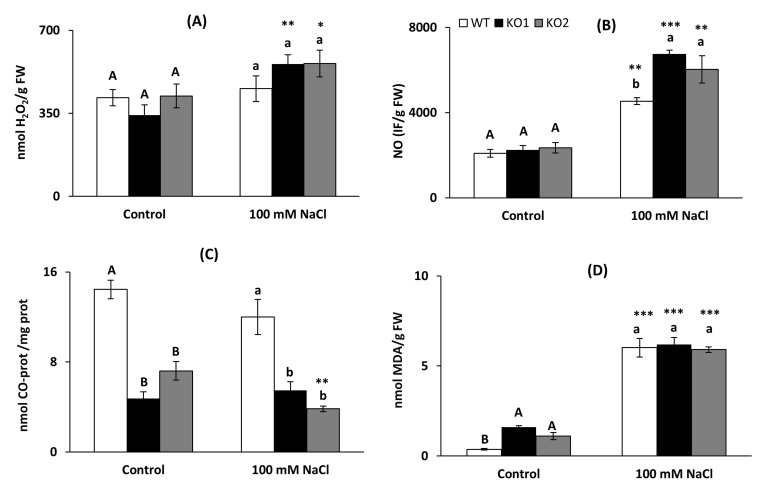
Oxidative stress markers. (**A**) Hydrogen peroxide, (**B**) nitric oxide (in arbitrary fluorescence units), (**C**) carbonyl (CO) proteins and (**D**) malondialdehyde (MDA) in leaves of *A. thaliana* wild type (WT) and two KO *Attrxo1* mutant lines grown in the absence (Control) and presence of 100 mM of NaCl. Data per fresh weight (FW) or mg proteins (prot.) are the mean ± SE of at least three independent experiments. Different letters indicate significant differences (*p* < 0.05) among genotypes in each condition according to the Tukey’s test, and the asterisks indicate significant differences of each genotype in salinity compared to the control condition using the *t*-Student’s test (*, **, *** at *p* < 0.05, *p* < 0.01 and *p* < 0.001, respectively).

H_2_O_2_ and NO contents were analyzed in WT and two *Attrxo1* mutant lines grown in control and saline conditions. No significant differences were found in H_2_O_2_ among genotypes in either control or saline conditions, although salinity increased levels in the mutant lines (Figure 5A). With regard to NO, *Attrxo1* mutants showed a higher content than the WT only in saline conditions, which provoked an increase in all genotypes (Figure 5B). 

Carbonylated proteins (CO-Proteins) and lipid peroxidation evaluated as malondialdehyde (MDA) level were analyzed as biomarkers of oxidative damage. Under both control and saline conditions, plants lacking *AtTRXo1* had less carbonylated proteins than WT (Figure 5C). Only in control conditions KO mutants presented higher MDA content than WT plants and salinity provoked a high increase in all genotypes (Figure 5D). 

### 2.4. Ascorbate, Glutathione and Nitrosoglutathione

**Figure 6:**
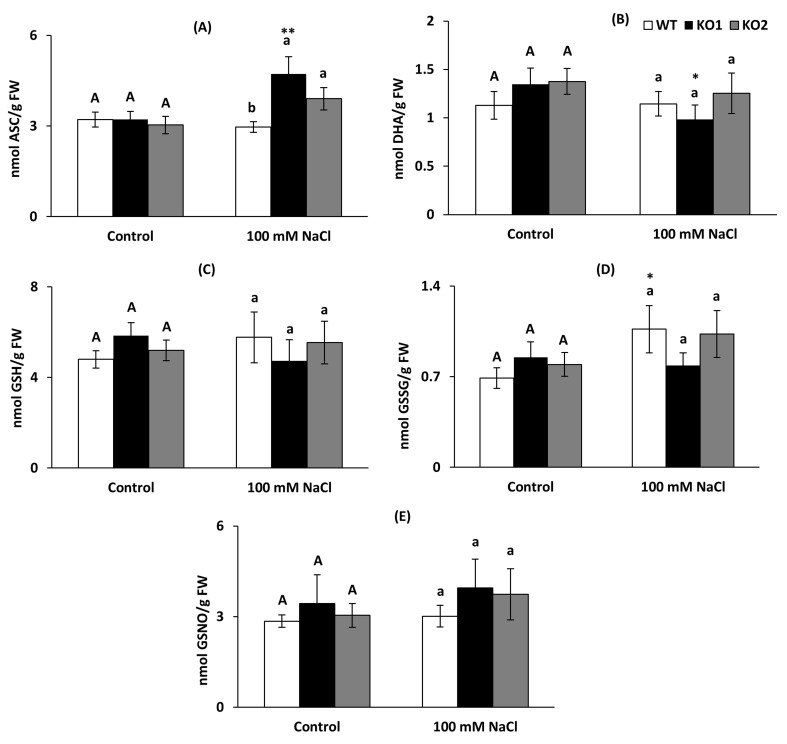
Ascorbate, glutathione and nitrosoglutathione content in leaves of *A. thaliana* wild type (WT) and two KO *Attrxo1* mutant lines grown in the absence (Control) and presence of 100 mM of NaCl. (**A**) Reduced ascorbate (ASC), (**B**) dehydroascorbate (DHA), (**C**) reduced glutathione (GSH), (**D**) oxidized glutathione (GSSG) and (**E**) nitrosoglutathione (GSNO) content per fresh weight (FW). Data are the mean ± SE of at least three independent experiments. Different letters indicate significant differences (*p* < 0.05) among genotypes in each condition according to the Tukey’s test, and the asterisks indicate significant differences of each genotype in salinity compared to the control condition using the *t*-Student’s test (*, ** at *p* < 0.05 and *p* < 0.01, respectively).

Antioxidant molecules involved in the Halliwell-Asada-Foyer cycle in their reduced and oxidized form (reduced glutathione GSH, reduced ascorbate ASC and oxidized dehydroascorbate DHA and glutathione GSSG) as well as the derived nitrosoglutathione (nitric oxide and glutathione reservoir) were quantified in the three genotypes under both control and saline conditions (Figure 6). None of these parameters presented significant changes among the different genotypes in both conditions, except for higher levels of ASC in the KO lines under salinity (Figure 6A). The only significant changes caused by salinity (asterisks in Figure 6) were an increase in ASC concomitant with a decrease in DHA in KO1 plants (Figure 6A,B) and an increase in GSSG in WT plants (Figure 6D).

The redox states of glutathione and ascorbate were determined as the percentage of the ratio of their reduced to their total contents, which are indicators of the rate of oxidation of these antioxidants and therefore an indirect variable of the level of oxidative stress. The ascorbate redox state tended to have an opposite trend in the KO plants to that observed in the WT plants, with an increase with salinity of the reduced form of this antioxidant in the mutant plants (Table 1). The glutathione redox state was very similar in all genotypes in both conditions (Table 1).

### 2.5. Hormone Content

**Figure 7:**
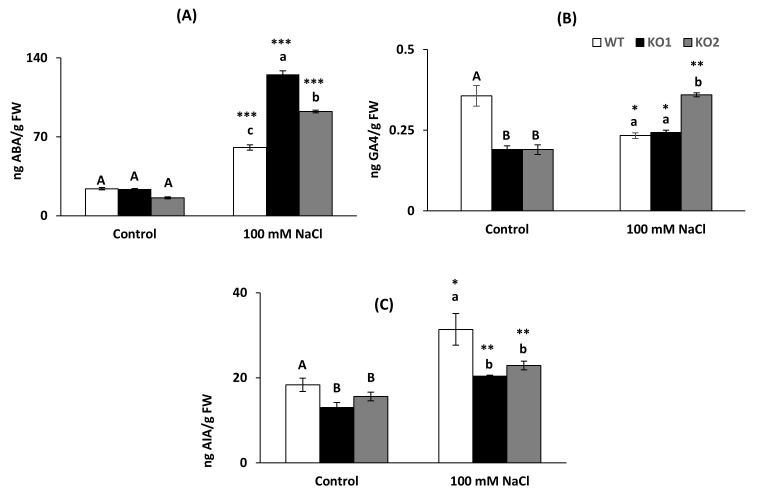
Hormone content in leaves of *A. thaliana* wild type (WT) and two KO *Attrxo1* mutant lines grown in the absence (Control) and presence of 100 mM of NaCl. (**A**) Abscisic acid ABA, (**B**) gibberellin GA4 and (**C**) indoleacetic acid IAA are expressed by fresh weight (FW). Data are the mean ± SE of at least three independent experiments. Different letters indicate significant differences (*p* < 0.05) among genotypes in each condition according to the Tukey’s test, and the asterisks indicate significant differences of each genotype in salinity compared to the control condition using the *t*-Student’s test (*, **, *** at *p* < 0.05, *p* < 0.01 and *p* < 0.001, respectively).

The hormones abscisic acid (ABA), indoleacetic acid (IAA) and the bioactive gibberellin GA4 were quantified in the three genotypes and in the two growth conditions. KO lines presented similar ABA content than WT plants under control condition but under salinity, mutants contained higher content (Figure 7A). The stress condition led to an increase in ABA in the three genotypes. GA4 levels were lower in the KO plants in control condition while under salinity the behavior of KO1 and KO2 was different, presenting KO2 a higher amount (Figure 7B). The *Attrxo1* mutant genotypes displayed lower levels of IAA than WT plants under both control and saline conditions (Figure 7C) and salinity increased the content in all the genotypes. 

### 2.6. Primary Metabolite Profiles

**Figure 8:**
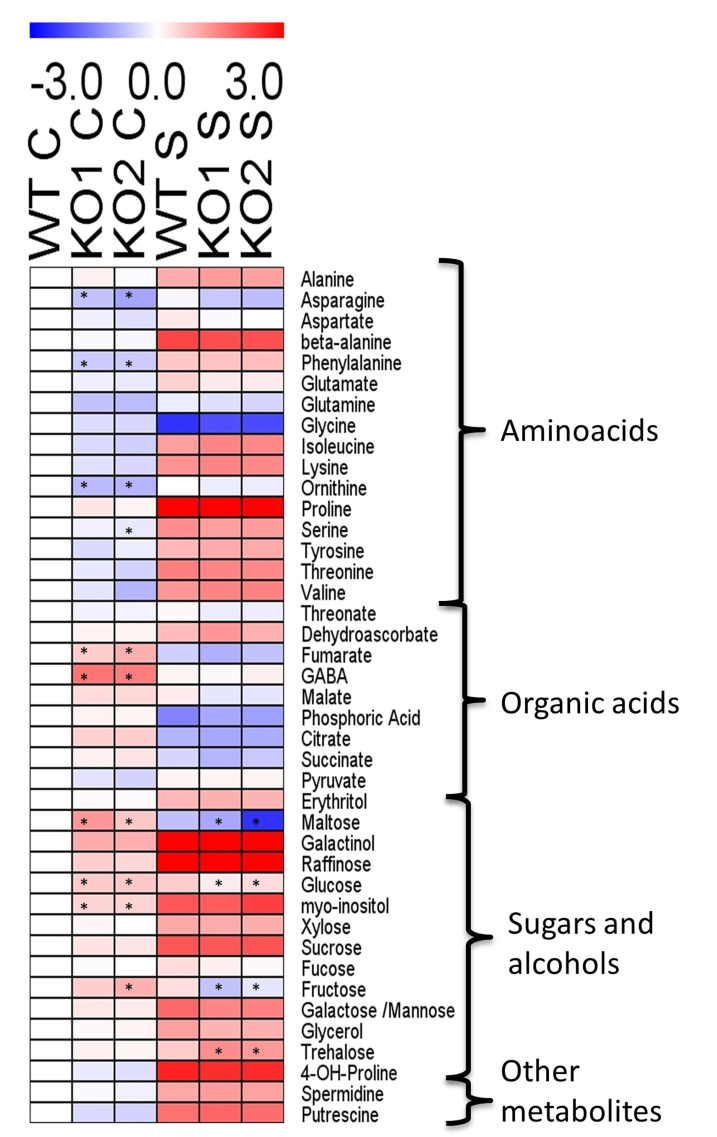
Heat map of relative levels of metabolites in leaves of *A. thaliana* wild type (WT) and two KO *Attrxo1* mutant lines grown in the absence (Control) and presence of 100 mM of NaCl. Relative metabolite levels in leaves of all lines under control and saline conditions normalized with respect to the mean value of WT plants under control condition. Fold-changes were log_2_ transformed (i.e., all metabolite levels from WT in control condition were 0). The red and blue colors represent the increases and decreases, respectively. The values represent the average from six samples and the asterisks indicate significant differences (*p* < 0.05) from the WT in each condition using the *t*-Student’s test. The statistically significant differences between control and salinity of each genotype are presented in Table S1.

Metabolite profiling analysis from the aerial part revealed that plants lacking *AtTRXo1* displayed higher levels of fumarate, GABA, glucose, maltose, galactinol and myo-inositol as well as lower levels of asparagine, phenylalanine and ornithine under controlled conditions as compared to WT (Figure 8, Table S1). Under saline conditions, KO mutants displayed significantly lower levels of fructose, glucose and maltose and higher levels of trehalose as compared to the WT plants. 

Regarding the effect of salinity only the levels of asparagine, aspartate, glutamine and threonine did not significantly change in any of the genotypes (Table S1). The level of glycine, phosphoric acid, citrate, fumarate, succinate and maltose decreased in all of the genotypes while the remaining measured metabolites increased in all genotypes under saline conditions.

### 2.7. Photosynthetic Parameters

**Figure 9:**
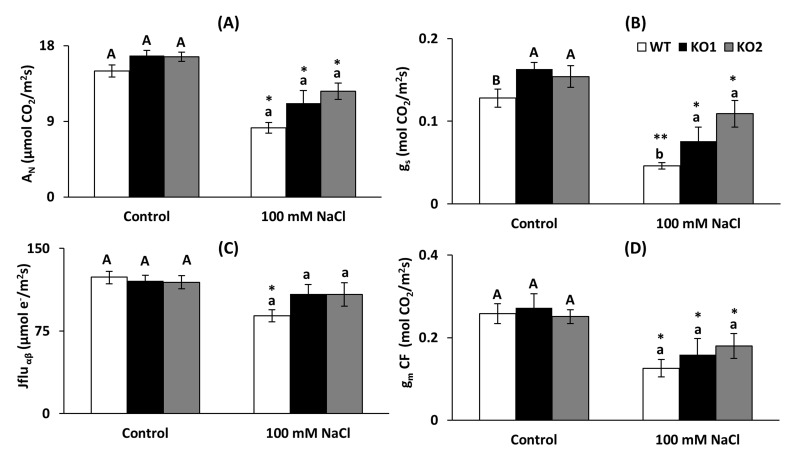
Photosynthetic parameters in leaves of *A. thaliana* wild type (WT) and two KO *Attrxo1* mutant lines grown in the absence (Control) and presence of 100 mM of NaCl. (**A**) Net photosynthetic assimilation (A_N_), (**B**) stomatal conductance to CO_2_ (g_s_), (**C**) electron transport rate (J_flu_) and (**D**) mesophyll conductance from curve-fitting (g_mCF_). Data are the mean ± SE of at least 9 replicates. Different letters indicate significant differences (*p* < 0.05) among genotypes in each condition according to the Tukey’s test, and the asterisks indicate significant differences of each genotype in salinity compared to the control condition using the *t*-Student’s test (*p* < 0.05).

Gas exchange parameters as net photosynthetic assimilation rate (*A*_N_), stomatal conductance (*g*_s_), electron transport rate (J_flu_) and mesophyll conductance estimation (which was calculated using two methods (variable *J* and curve-fitting) being strongly correlated across genotypes and treatments (*r* = 0.93; *p* = 0.007), hence only *g*_mCF_ is showed) were mostly affected by salt stress decreasing in all genotypes (Figure 9A–D, respectively), except J_flu_ which was maintained in the KO lines (Figure 9C). Only stomatal conductance was different among genotypes, being higher under both conditions in the KO than WT plants (27% and 20%, respectively, Figure 9B). 

**Figure 10:**
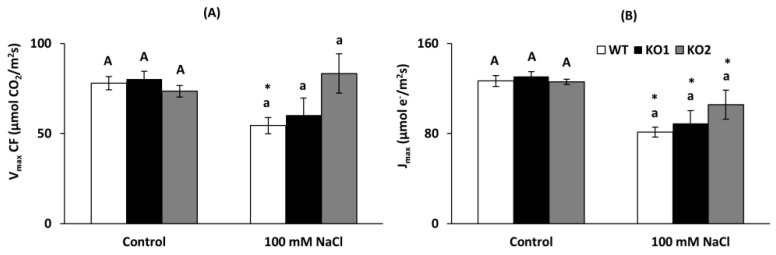
Photosynthetic parameters in leaves of *A. thaliana* wild type (WT) and two KO *Attrxo1* mutant lines grown in the absence (Control) and presence of 100 mM of NaCl. (**A**) Maximum velocity of Rubisco carboxylation (V_cmax_) and (**B**) maximum electron transport rate (J_max_). Data are the mean ± SE of at least 9 replicates. Different letters indicate significant differences (*p* < 0.05) among genotypes in each condition according to the Tukey’s test, and the asterisks indicate significant differences of each genotype in salinity compared to the control condition using the *t*-Student’s test (*p* < 0.05).

In addition, maximum velocity of Rubisco carboxylation (V_cmax_) and maximum electron transport rate (J_max_) were similar in all the genotypes (Figure 10A,B), although salinity exerted a different effect only in V_cmax_ since in the mutants this parameter did not decrease so much as in the WT plants. J_max_ however decreased similarly in all genotypes when they grew in the presence of the salt.

## 3. Discussion

Photosynthesis is one of the most affected processes under salinity partially due to the osmotic effects and the oxidative stress associated therewith [36,37]. As a result, the redox and antioxidant metabolisms are altered and contribute to the stress response. In this context, the lack of a protein involved in redox regulation such as TRX*o*1 could affect the stress response and indeed, recent studies on KO *Attrxo1* mutants grown in salinity conditions have revealed a role for this TRX*o*1 in responding to this adverse situation [29]. These mutants have been previously reported as Knock-out mutants and the molecular data analysis showed the lack of expression of both lines [28] by PCR analysis and the lack of expression in KO1 line and only a very low residual expression in KO2 when measured by qPCR [33]. Loss of function of these mutants has allowed to corroborate a role for the TRX system in regulating key mitochondrial processes not only under control but also under stress conditions [25,29], with the description of a lack of extreme phenotype due possibly to the existence of redundant or compensatory activities of other TRXs or GRXs. Moreover, some differences have been previously reported between both KO lines in different parameters, not only of growth but also antioxidant components, metabolites, oxidative stress parameters in response to salinity and drought [10,27,28,29,33]. Differences among lines are not visualized as commented above, but the measurement of a high number of samples have shown some significant differences in growth parameters as leaf area/plant between KO1 and KO2 lines and in the number of leaves between WT and KO1 plants. As we previously reported, these and other differences between both mutants could be due in part to the residual *AtTrxo1* gene expression showed by KO2 plants. More recently, it has been shown that *Attrxo1* mutants displayed a reorganization of the mitochondrial antioxidant system, sugar metabolism and cytochrome oxidase pathway, possibly through the regulation of target proteins involved in these processes [10]. All these changes probably allowed the mutants to cope with the oxidative stress produced by the saline conditions. Interestingly, *Attrxo1* mutants displayed higher stomatal density together with a lower pore size in control conditions as well as a lower water loss and reduced stomatal pore size under salinity [29]. In addition to our previous studies on TRX*o*1 function in salinity adaptation, the importance of the mitochondrial NTR/TRX system under drought episodes was reported in both *Attrxo1* mutant plants which presented diverse and complex responses as a result of both single and repetitive drought episodes [28]. In this study, the analysis of the increased stomatal conductance following drought recovery also pointed to a TRX*o*1 redox regulation of stomatal function, although stomatal size or aperture were not reported. All these previous results suggest that TRX*o*1 could be involved in stomatal development and aperture under both normal and saline conditions, probably through the redox regulation of key target proteins involved in the process, possibly influencing the functioning of the photosynthetic process. In order to further analyze this, we have studied the impact of the decreased level of TRX*o*1 of mutant genotypes, in the photosynthetic process of plants grown in the absence (control) and presence of 100 mM NaCl under short day and moderate light conditions (300 μmol·m^−2^·s^−1^ PAR) which are more suitable for the analysis of certain photosynthetic parameters as the mesophyll conductance. Growth parameters were significantly reduced by salinity in WT and the two lines of KO *Attrxo1* mutants. Nevertheless, the lower leaf area together with higher number of leaves observed in KO1 plants indicate a growth alteration related to the *TRXo1* gene expression level. Similar to this growth effect, reduced fresh weight has recently been reported in the *Attrxo1* mutants grown under short-day conditions and lower growth light intensity (150 μmol·m^−2^·s^−1^ PAR) [27]. On the other hand, a lack of significant changes in growth parameters was reported under both, control or saline conditions in plants grown under this lower light intensity but long day photoperiod [29] (Table S2 for data comparison). All these results denote that phenotypic changes caused by *TRXo1* alterations are considerably dependent on the growth conditions, which could have also influenced the plant redox and metabolic responses.

### 3.1. Changes in Carbon Rather Than in Redox Metabolism Are Related to Leaf Stomatal Development and Aperture in trxo1-Altered Plants under Control Conditions

In our experimental conditions, total stomatal density was similar among genotypes, but the number of mature stomata was lower in KO plants although they were of a higher size, suggesting an involvement of TRX*o*1 in the stomatal development, which could be attributed to a disturbance of the cell redox state under control conditions. The relationship between cell redox changes and the development of stomata still remains relatively unexplored although some experimental evidence have been reported: MAPK3 and 6 are mitogen activated protein kinases (MAPK) involved in stomatal development although the mechanisms by which they are activated/inactivated and regulate the different stages of stomatal development are relatively unknown [38]. Recently, a dual phosphatase, MAP kinase phosphatase 1 (MPK1) has been identified to promote the transition to the stomatal cell destination by controlling the activation of MAP kinases at the initial stages of development [39]. In this context, the existence of a redox control by H_2_O_2_ on certain MAPKs has been described [40], which may imply the possible control of the process by redox proteins as TRXs. An interesting example of redox control on stomatal density that also involves proteins of the TRX/PRX system is that of the proteins SDD1 (Stomatal Density and Distribution1-1) and EPF1 (epidermal pattering Factor1) of plasma membranes, which are negative regulators of the stomatal development [41]. The mutant sdd1-1 has a high stomatal density as well as NADPH thioredoxin reductase NTRC mutants, which displayed a repression of the SDD1 and EFP1 genes when grown on a short day [42]. 

Besides stomatal development, there is evidence for the existence of a redox control for stomatal opening. The activity of ionic channels can be modified by H_2_O_2_ thus acting as redox sensors [43], together with RLKs kinases rich in Cys residues (CRKs) [44] that have been involved in the control of the basal opening and response to stimuli. As another example, ASPG1 (aspartic protease in guard cell), a protein involved in ABA response under drought stress, has been reported as another potential Trx h target in *Brasicca napus* [45]. In the present study, a higher stomatal pore size was observed in our KO *Attrxo1* mutants under control conditions pointing TRX*o*1 as involved in the process, although no changes in H_2_O_2_ and NO levels were observed despite their increased lipid peroxidation and decreased CO-proteins. Moreover, neither changes in antioxidant molecules involved in the Halliwell-Asada-Foyer cycle (reduced glutathione and reduced ascorbate) nor in nitrosoglutathione were observed in the mutants. Therefore, at least at foliar levels we did not find any evidence of a redox-related mechanism by these components controlling stomatal aperture. While the role of oxidative modifications of ionic channels is well documented in animal systems, the ability to “sense” ROS of these and other possible sensors in plants remains to be demonstrated.

The larger size of the stomata, as well as a higher pore size in the KOs are contributing to the observed higher stomatal conductance in these plants, taking also into account the similar stomatal density. This higher aperture contributes to the total pore area in the leaves and consequently to the total gas exchange. It is well established that the maximum conductance is related to stomatal density and stomata size as well as the hydraulic conductance of the leaf although 3D morphology of the stomata and mesophyll structure and air space have been recently proposed to contribute [46,47,48]. On the other hand, our detailed photosynthetic analysis revealed that *TRXo1* alteration did not induce any significant changes in other photosynthetic parameters. This is in agreement with lack of changes in the photosynthetic performance of these mutants under control conditions reported in previous studies [27,28]. Similarly, it has been reported that no simple relationship exists between stomatal density, photosynthesis and yield, and moreover an increased stomatal conductance in adapted plants to similar conditions of light intensity than ours, was described to not correlate with substantial changes in the assimilatory performance in high stomatal density *Atsdd1-1* mutants [49]. The underlying mechanism for the mentioned higher stomatal aperture is probably not related to an altered signaling by ABA, since its content was similar in all genotypes under non-stress conditions. On the other hand, the higher levels of fumarate observed in the KO mutant can be related with the stomatal opening [50,51]. Different studies have reported the effect of TRX*o*1 mutation on the up regulation of respiratory metabolism thus affecting fumarate/malate levels [25,27]. The precise direction of changes in malate/fumarate levels differed among the present and previously published studies, but this can be due to different day-time harvesting and growth light conditions [27]. The higher levels of GABA observed in the *Attrxo1* mutants in the present study suggest an altered TCA cycle regulation, since the GABA shunt can replenish succinate to the TCA cycle. In agreement, higher ^13^C label redistribution to GABA in *Attrxo1* mutants has been suggested to support an increased TCA cycle flux from succinate to fumarate or malate [27].

### 3.2. Changes in Carbon and Redox Metabolism Are Coordinated with the Hormonal Response for the Better Adaptation of trxo1 Mutants to Saline Stress 

The inhibition of photosynthesis by saline stress can be due to the closure of stomata and the limitation of intracellular CO_2_ levels [52], but also to a decrease in PSII activity and electron transport [53]. In this study, all genotypes presented a typical response to the saline condition. The observed decreases in the measured photosynthetic parameters are in accordance with what is described in the literature in terms of response to both saline stress and drought [54,55,56], although in the mutants some of the observed decreases were not significant. This response usually occurs under moderate stress, where there is a stomatal closure, decreased g_s_ and consequently A_N_ but to a lesser extent. When the stress is intense, a decrease in g_m_ and all photosynthetic parameters usually occurs [57,58]. The observed decrease in g_s_ under salinity (about 50%) falls within the mild stress range for photosynthesis [3,59,60]. Hence, *g*_m_, *V*_cmax_, *J*_max_ and *J*_flu_ experienced only moderate declines. While statistically it appears that the lack of *AtTRXo1* caused maintenance of the electron transport rate (*J*_flu_), changes were too small to induce any significant variation in net CO_2_ assimilation. 

Previous studies in Arabidopsis *trxo1* mutant plants subjected to long-term 100 mM NaCl treatment denote a lack of visible phenotype and a reorganization of redox and carbon metabolism as an acclimation to the long-term salinity treatment [10,29]. Similarly, a redox imbalance of the *Attrxo1*-altered plants under salinity has also been observed in the present study. Signaling components including ROS and generated oxidative products are fundamental in producing a response of the plants and their adaptation to stressful conditions. In this context, the antioxidant and redox balance system that includes the TRXs located in the different cell compartments, play an essential role in such response [1,7,61]. The analysis of the oxidative and antioxidant status of our plants revealed that compared with WT plants, the *trxo1*-altered plants displayed higher increase in the level of H_2_O_2_ and higher levels of NO, implying that the deficiency of *TRXo1* produced an imbalance in the oxidative state of these plants under saline conditions. We found similar results in a previous work with this KO mutant, although changes induced by salinity and differences among genotypes were less pronounced in plants grown at long photoperiod and lower light intensity [29]. Moreover, the antioxidant system including SOD, catalase and components of the ASC-GSH cycle were previously found altered in the *trxo1* mutants grown in the long-photoperiod, thus again denoting the influence of the different light conditions in the response of the mutants to salinity (Table S2). The higher levels and redox state of ASC in the *trxo1* mutants observed here could collaborate in the adaptation that these plants showed to the saline stress situation. Accordingly, it has been described that lower ascorbate content in *vitc* mutants caused an increase in sensitivity by the oxidative stress associated with saline treatments [62]. All together the results denote that the decreased in TRX*o*1 influences the final oxidative/antioxidant status of certain parameters depending on the growth light conditions, thus playing a role in the adaptation of plants to different stress conditions as previously described in the same mutant lines upon drought grown under short photoperiod but less light intensity [28].

Phytohormones are key components in the signaling process that direct and regulate plant growth and development with a crucial role in the response to stress situations such as salinity, modulating adaptation [63]. The limiting step in ABA biosynthesis is that carried out by the 9-cis-epoxy carotenoid dioxigenase (NCED) that generates xantoxine which is oxidized to ABA [64]. The transcriptional regulation of *NCED* in leaves depends on light and suggests a relationship with photosynthesis and probably redox signaling [65], although it is not very well known whether is also subjected to post-translational regulation. A circumstantial relationship between ABA biosynthesis and chloroplast redox status is established through ascorbate, as *vtc1* mutants with low ascorbate are correlated with ABA increments in leaves [66]. Another possible relationship is established through the Mg chelatase involved in chlorophyll synthesis, plastid-nucleus retrograde signaling and ABA perception. This Mg chelatase is redox regulated by TRX *f* and TRX *m* [67], and in fact, our mutants present a slightly higher content in chlorophyll in control conditions, pointing to a possible redox regulation of the synthesis, although we cannot exclude the possibility that the observed slight increase in chlorophyll could be biologically not significant. Moreover, *Attrxo1* mutant lines showed much higher increase in ABA levels under salinity, which could indicate that redox imbalance was sensed in this stress situation thus affecting the hormone content. Increased levels of ABA under salinity have been associated to the regulation of several stress-responsive genes, the reduction of Cl^−^ and Na^+^ levels as well as the increase in proline and sugars levels [68]. Furthermore, ABA induces stomatal closure and NO is a key molecule involved in this signaling pathway controlling stomatal movements. The NO levels were induced under salinity up to higher levels in the *trxo1* mutant as compared to WT plants, thus suggesting, together with the observed increased ABA levels, that these mutants probably display a stronger response of the ABA signaling pathway inducing stomatal closure. This could be related to the fact that mutant plants display a higher stomatal density and therefore require a higher leaf response of stomatal closure in order to control the water loss during salinity stress. In addition to stomatal movement, ABA also affects stomatal development at multiple levels. In Arabidopsis a high stomatal density has been associated to mutants defective in ABA metabolism or signaling [69] and ABA negatively regulates initiation of stomatal development in Arabidopsis cotyledons [70].

In that sense, the increase in stomatal density in both altered-*trxo1* mutants under salinity occurs with elevated ABA content, which seems to promote and not repress stomatal formation, although we cannot ignore the possibility that these *trxo1* mutants have a defective ABA sensing and/or signaling. Interestingly, there is evidence showing that sucrose metabolism can induce stomatal closure and that sucrose-induced stomatal closure is ABA-dependent [71,72]. While sucrose levels were similar among genotypes, significantly lower levels of glucose and fructose suggest a higher leaf glycolytic activity. Sucrose catabolism sustains glycolysis, the tricarboxylic acid (TCA) cycle and glutamine biosynthesis, which can promote light-induced stomatal opening [73]. In addition, both *trxo1*-altered mutants under salinity displayed higher levels of trehalose which metabolism is closely related with the responsiveness of guard cells to ABA and the control of stomatal conductance [72,74]. On the other hand, some recent data have reported the role of IAA in stomatal development. Our data fit well with that described in this regard, especially in the KO mutant under salinity, with less IAA content and higher stomatal density.

In summary, sugar changes observed in parallel to increases in ABA and NO suggests that *trxo1*-altered plants display a higher leaf stomatal response under salinity. The increased carbon flux from glycolysis towards the TCA cycle previously observed could be the cause for an increased ABA signaling required to close the higher number of stomata in *trxo1*-altered plants under salinity conditions.

## 4. Materials and Methods 

### 4.1. Plant Material and Growth

*Arabidopsis thaliana* ecotype Columbia (Col-0; the wild type, WT) and two T-DNA insertion mutants [described as knock-out (KO) *Attrxo1*: SALK_143294C (KO1) and SALK_042792 (KO2)] obtained from the European Arabidopsis Stock Centre (NASC, http://Arabidopsis.info/) [28,33] were used in this work. 

Plants (one per pot) were grown in substrate (peatsoil:perlite:vermiculite (2:1:1 *v*/*v*/*v*) under controlled conditions of light (300 μmol·m^−2^·s^−1^ PAR), photopheriod (8/16 h light/dark), relative humidity (65–70%) and temperature (23/18 °C light/dark). The response of WT and KO plants to salt stress was studied in 33-day old plants exposed to Hoagland solution (control) and 100 mM NaCl three times a week for 21 days.

### 4.2. Growth Analysis

Growth of WT and KO lines in the absence and presence of 100 mM NaCl was evaluated by measuring number of leaves, aerial part’s fresh weight, rosette diameter and foliar area in 33-day old plants.

### 4.3. Stomatal Characterization

Nail polish imprints were taken from the abaxial surface of fully developed leaves from WT and KO *Attrxo1* plants as described before [75]. Stomatal densities were determined by light microscopy as previously described [29], with four independent counts carried out on 5 independent leaves (one leaf per plant). Three independent experiments were carried out (*n* > 2000 stomata per genotype and treatment). Total stomata included immature and mature structures, considering mature stomata the ones presenting fully developed guard cells and pore, as previously described [34,35]. Moreover, the length of the guard cells was measured in these peelings. To characterize the stomatal opening, early in the light period (with most of the stomata open), 5 leaves for each genotype and treatment were separated by cutting the base of the petiole with a scalpel. The epidermis was removed from the abaxial surface with the aid of adhesive tape. The obtained peeling was dyed with 0.5 µM rhodamine 6G for 5 min in darkness for a better visualization and measurement of the stomatal pore [75]. After washing the peeling with MES-KOH buffer to remove the excess of dye, epifluorescence allowed to contrast the guard cells and analyze the stomatal opening using a Leica CTR6 microscope (40X objective with excitation light at 541 nm and emission light at 565 nm). The stomatal opening index was calculated by division of the width of the stomata pore by length. Three independent experiments were carried out with at least 500 stomata analyzed per genotype and treatment. The image analysis was carried out with the ImageJ software (https://imagej.nih.gov/ij/).

### 4.4. Chlorophyll Content

For the measurement of chlorophyll, the SPAD-502 m has been used, which produces relative values that are proportional to the amount of chlorophyll present in the leaves, expressing the values as nmol·cm^−2^ chlorophyll as previously described [76]. 

### 4.5. Hydrogen Peroxide, Lipid Peroxidation, Carbonyl Proteins and Nitric Oxide Contents

Leaf samples (1 g) were homogenized in a mortar with 2 mL of 50 mM potassium phosphate pH 7.8; 0.1 mM EDTA; 0.2 % Triton X-100; 1% PVP and protease inhibitors 1X (cOmplete, Roche™, Mannheim, Germany).

The H_2_O_2_ content was measured by the ethanol ferrous oxidation−xylenol orange method [77] (e-FOX) and lipid peroxidation was determined measuring the substances reacting with thiobarbituric acid [78] as previously described [29]. Carbonyl protein content was measured as carbonyl content [79] as previously reported [80]. The total soluble protein content was quantified using Bradford’s method [81]. Nitric oxide (NO) content was analyzed by fluorimetry using 4,5-diaminofluorescein (DAF-2) as previously described [82]. 

### 4.6. Hormone Analysis

The content of ABA, IAA and GA4 of leaves was analyzed by UHPLC-mass spectrometry (Q-Exactive, ThermoFisher Scientific, Madrid, Spain) in samples of 50 mg fresh weight (Plant Hormone Quantification Service of the Institute of Molecular and Cellular Plant Biology, CSIC-UPV, Valencia, Spain). Samples were ground in liquid N_2_ and lyophilized. Material was extracted with 80% methanol-1% acetic acid containing internal deuterium-labelled standards and after vacuum evaporation, the dry residue was dissolved in 1% acetic acid and passed through reverse phase Oasis HLB column and dissolved in 5% acetonitrile-1% acetic acid [83]. Hormones were separated by reverse phase UPHL chromatography and analysed with a Q-Exactive mass spectrometer (Orbitrap detector, ThermoFisher Scientific, Madrid, Spain) by targeted Selected Ion Monitoring. Hormone concentration was determined using embedded calibration curves and the Xcalibur v4.1 SP1 build and TraceFinder programs. All the processing and analysis has been carried out at the Plant Hormone Quantification Service, IBMCP-UPV, Valencia, Spain. 

### 4.7. Metabolite Profiling

Leaf samples were grinded in liquid nitrogen and 50 mg of frozen-powdered tissue were homogenized and analyzed by mass spectrometry as previously described [10]. 

### 4.8. Ascorbate, Glutathione and Nitrosoglutathione

Frozen plant tissue (500 mg) was ground in liquid N_2_ and homogenized with 1 mL of cold (4 °C) extraction solution (5% m-phosphoric acid (*w*/*v*), 1 mM EDTA and 0.1% formic acid), supplemented with 1% polyvinyl-polypyrrolidone (*w*/*v*) just before use. Analyses were carried out by mass spectrometry as previously described [29]. 

### 4.9. Gas Exchange and Chlorophyll Fluorescence

An open gas exchange system with a coupled fluorescence chamber of 2 cm^2^ (Li-6400XT; Li-Cor Inc., Lincoln, NE, USA) was used for simultaneous measurements of gas exchange and chlorophyll fluorescence. CO_2_ leakage in the leaf-gasket interface was determined and used for the accurate estimation of all photosynthetic parameters [84]. In 9–11 leaves from different individuals of each line and treatment, light-saturated net assimilation (*A*_N_), stomatal conductance to CO_2_ (*g*_s_), substomatal CO_2_ concentration (*C*_i_) and photochemical yield of photosystem II (φ_PSII_) were obtained after 20–30 min at ambient CO_2_ of 400 μmol·mol^−1^, photosynthetic photon flux density (PPFD) of 1500 μmol·m^−2^·s^−1^ (90 % red and 10 % blue light), block temperature of 25 °C and chamber relative humidity of 50–70 %. Subsequently, *A*-*C*_i_ curves were obtained by first lowering ambient CO_2_ from 400 to 50 μmol CO_2_·mol^−1^ in 7 steps and then increasing from 400 to 1500 μmol CO_2_·mol^−1^ in 8 steps with a stabilization time at each CO_2_ concentration of 3–4 min. In addition, light curves were performed under non-photorespiratory conditions (~2% O_2_) by connecting the Li-6400XT to a N_2_ tank coupled with a custom-made air humidifier. Light curves consisted of 13 steps of decreasing PPFD from 1500 to 0 μmol·m^−2^·s^−1^. Dark respiration (*R*_d_) was measured after >30 min under dark conditions and used as a proxy of light respiration for parameter estimation. Electron transport rate (*J*_flu_) was estimated as J_flu_ = φ_PSII_ × PPFD × αβ where αβ is the product between leaf absorbance (α) and the electron partitioning between PSI and PSII [85] and was estimated as previously detailed [86]. The curve-fitting method [87,88] was used to estimate mesophyll conductance to CO_2_ (*g*_mCF_), maximum velocity of Rubisco carboxylation (*V*_cmax_) and maximum electron transport rate (*J*_max_) as previously described [89]. Photocompensation point without respiration (Γ*) and its temperature dependencies (*c*, Δ*Ha*) for *Arabidopsis thaliana* were taken from previously reported data [90]. 

### 4.10. Statistical Analysis

The experiments were conducted in a completely randomized design. Each experiment was repeated at least three times with three replicates per treatment for each genotype and at least four plants per replica. Two different statistical analysis were applied as previously described [10]: one for differences among genotypes in each condition (capital letters for control and lower-case letters for saline conditions) with an analysis of the variance (ANOVA, one factor) using the Tukey’s test (*p* < 0.05), and another test for the salt effect in each genotype (asterisk when significant differences under salinity compared with control condition) using Student’s *t*-test (*p* < 0.05). IBM SPSS Statistics 20 program (Statistical Package for Social Sciences, 2011, IBM, Armonk, NW, USA) and JMP^®^, v12.1.0 (SAS Institute Inc., Cary, NC, USA, 1989–2007) were used for the data analysis.

## 5. Conclusions

The differences in stomatal density, development, size and aperture between WT and KO *Attrxo1* mutants observed under control and saline conditions suggest a role of TRX*o*1 in stomatal development and functioning. Under control conditions, stomatal responses appear to be more related to changes in organic acid metabolism than to ABA and redox changes. On the other hand, the elevated NO and ABA contents in the mutants under salinity were not correlated with a higher stomatal closure but are probably the consequence of a higher stomatal number responding to the salinity induced stomatal closure. In parallel to ABA and NO changes, the different patterns of glucose, fructose and trehalose among genotypes under salinity denotes that the coordination between ABA and sugar metabolism for the control of the stomatal closure is altered in the *trxo1* plants. Any of these changes did not ultimately affect the photosynthetic process and allowed the maintenance of the energy balance necessary for the growth in the stress situation of the plants with decreased levels of *AtTRXo1.* These results point out to an expansion of the role of this TRX*o*1 through the redox regulation of its different target proteins involved in numerous processes involved in the saline stress response, including stomata development and behavior. 

## Figures and Tables

**Table 1:** Redox state of ascorbate and glutathione in leaves of *A. thaliana* wild type (WT) and two lines of KO *Attrxo1* plants grown under control conditions and in the presence of 100 mM of NaCl. Redox state was calculated as ASC/ASC+DHA and GSH/GSH+GSSG percentages.

	WT	KO1	KO2
	Redox State ASC/ASC + DHA (%)		
Control	74.0	70.5	68.8
100 mM NaCl	72.1	82.8	75.6
	Redox State GSH/GSH + GSSG (%)		
Control	87.4	87.3	86.7
100 mM NaCl	84.4	85.7	84.3

## Data Availability

Data is contained within the article or supplementary material.

## Supplementary Materials

Supplementary materials can be found at https://www.mdpi.com/1422-0067/22/3/1063/s1. Figure S1: Arabidopsis plants of 33 days old representative of the genotypes WT and two KO *Attrxo1* lines grown in the absence (Control) and presence of 100 mM NaCl for 21 days, Table S1: Relative levels of metabolites in leaves of *A. thaliana* wild type (WT) and two KO *Attrxo1* mutant lines grown in the absence (C) and presence of 100 mM of NaCl (S). The values represent the standard mean error of 6 standardized biological samples to the mean value in the WT in each condition. Numbers in bold indicate significant differences (*p* < 0.05) with respect to the control status of each genotype and asterisks indicate significant differences (*p* < 0.05) relative to the WT in each condition using the t-Student’s test, Table S2: Percentage (%) of increase (↑) or decrease (↓) of different parameters in two *Attrxo1* mutant lines (KO1 and KO2) related to wild type (WT) values of plants grown in the absence (Control) and presence (100 mM) of NaCl. Results belong to similar parameters measured in the same lines but in different conditions: 150 μmol·m^−2^·s^−1^ PAR, long photopheriod (16 h light/8 h dark) as described in Calderón et al. (2018) [29] and 80 μmol·m^−2^·s^−1^ PAR, long photopheriod (16 h light/8 h dark) and grown in agar plates as reported in Sánchez-Guerrero et al. (2019) [10]. Only significant differences are presented when compared to WT in each condition (*p* < 0.05) using the t-Student’s test. ns: not significant.

## Abbreviations

ABA	Abscisic acid
*A*_N_	Net photosynthetic assimilation rate
ASC	Ascorbate, reduced form
ASPG1	Aspartic protease G1
CO proteins	Carbonyl proteins
DAF-2	4,5-diaminofluorescein
DHA	Dehydroascorbate
EPF1	Epidermal pattering Factor1
FW	Fresh weight
GA	Gibberellin
*g*_m_	Mesophyll conductance
GRX	Glutaredoxin
*g*_s_	Stomatal conductance
GSH	Glutathione, reduced form
GSNO	Nitrosoglutathione
GSSG	Glutathione, oxidized form
IAA	Indoleacetic acid
J_flu_	Electron transport rate
J_max_	Maximum electron transport rate
KO	Knock-out
MAPK	Mitogen activated protein kinases
MDA	Malondialdehyde
MES	4-Morpholineethanesulfonic acid
NO	Nitric oxide
NTRC	NADPH thioredoxin reductase
MPK1	MAP kinase phosphatase 1
PRX	Peroxiredoxin
CRKs	RLKs kinases rich in Cys residues
PVPP	Polyvinyl-polypyrrolidone
RNS	Reactive nitrogen species
ROS	Reactive oxygen species
SDD1	Stomatal Density and Distribution1-1
TCA	Tricarboxylic acid
TRX	Thioredoxin
V_cmax_	Maximum velocity of Rubisco carboxylation
WT	Wild type
