# Supplementary material for: Decreased Levels of Thioredoxin o1 Influences Stomatal Development and Aperture but Not Photosynthesis under Non-Stress and Saline Conditions
Source: Int J Mol Sci. 2021 Jan 21;22(3):1063. doi: 10.3390/ijms22031063 (PMC7865980; doi:10.3390/ijms22031063)
Supplement: Supplementary file 1 [file ijms-22-01063-s001.zip › Supplementary Table S1.pdf]

Supplementary Table 1. Relative levels of metabolites in leaves of A. thaliana wild type (WT), and two KO Attrxo1 mutant lines grown under control (C) conditions and in the presence of 100 mM of NaCl (S). The values represent the standard mean error of 6 standardized biological samples to the mean value in the WT in each condition. Numbers in bold indicate significant differences (P<0.05) with respect to the control status of each genotype and asterisks indicate significant differences (P<0.05) relative to the WT in each condition using the t-Student's test.

**Metabolite**	**WT C**	**KO1 C**	**KO2 C**	**WT S**	**KO1 S**	**KO2 S**
Alanine	1±0.05	1.13±0.06	0.97±0.01	**1.98±0.18**	**2.25±0.05**	**2.17±0.05**
Asparagine	1±0.15	0.61±0.16*	0.48±0.02*	0.93±0.18	0.64±0.03	0.59±0.03
Aspartate	1±0.09	0.88±0.1	0.77±0.05	1.22±0.21	0.96±0.15	1.00±0.06
beta-alanine	1±0.09	0.95±0.03	0.93±0.07	**4.63±0.35**	**4.26±0.15**	**4.18±0.11**
Phenylalanine	1±0.15	0.66±0.04*	0.66±0.05*	**1.58±0.19**	**1.65±0.08**	**1.70±0.01**
Glutamate	1±0.06	0.88±0.11	0.84±0.08	**1.47±0.20**	**1.20±0.18**	**1.19±0.19**
Glutamine	1±0.15	0.61±0.10	0.58±0.07	0.86±0.11	0.77±0.15	0.71±0.20
Glycine	1±0.09	0.76±0.11	0.72±0.06	**0.19±0.01**	**0.24±0.02**	**0.23±0.04**
Isoleucine	1±0.14	0.74±0.03	0.68±0.01	**2.20±0.22**	**2.71±0.15**	**2.66±0.16**
Lysine	1±0.11	0.78±0.02	0.72±0.03	**2.46±0.36**	**2.77±0.24**	**2.68±0.03**
Ornithine	1±0.11	0.57±0.06*	0.55±0.05*	1.01±0.12	**0.86±0.05**	**0.87±0.14**
Proline	1±0.34	1.23±0.34	1.10±0.21	**93.70±3.10**	**66.26±8.18**	**63.42±6.20**
Serine	1±0.02	0.89±0.04	0.83±0.03*	**2.53±0.21**	**2.20±0.11**	**2.25±0.09**
Tyrosine	1±0.17	0.74 ±0.02	0.86±0.01	1.79±0.36	**2.01±0.23**	**2.00±0.26**
Threonine	1±0.02	0.83±0.04	0.70±0.02	**2.85±0.19**	**2.71±0.11**	**2.67±0.24**
Valine	1±0.08	0.81±0.04	0.55±0.03	**2.36± .24**	**2.73±0.12**	**2.79±0.27**
Threonate	1±0.07	0.90±0.08	0.91±0.05	1.06±0.17	0.86±0.09	0.87±0.08
Dehydroascorbate	1±0.24	1.12±0.10	1.10±0.23	1.74±0.23	**2.36±0.18**	**1.92±0.21**
Fumarate	1±0.10	1.52±0.21*	1.92±0.33*	**0.68±0.08**	**0.53±0.04**	**0.61±0.31**
GABA	1±0.08	3.14±0.39*	2.86±0.31*	1.11±0.04	**1.06±0.10**	**1.14±0.26**
Malate	1±0.19	1.34±0.12	1.39±0.22	1.18±0.25	**0.82±0.13**	**0.81±0.11**
Phosphoric acid	1±0.15	1.11±0.12	1.09±0.15	**0.36±0.07**	**0.49±0.08**	**0.46±0.07**
Citrate	1±0.27	1.47±0.18	1.52±0.22	0.54±0.08	**0.49±0.10**	**0.51±0.12**
Succinate	1±0.06	1.13±0.11	1.25±0.43	**0.71±0.09**	**0.55±0.06**	**0.64±0.02**
Pyruvate	1±0.10	0.80±0.06	0.70±0.03	1.09±0.07	**1.12±0.05**	**1.09±0.09**
Erythritol	1±0.07	1.05±0.07	1.04±0.06	**1.80±0.17**	**1.92±0.16**	**1.86±0.32**
Maltose	1±0.05	2.33±0.34*	1.59±0.45*	**0.60±0.03**	**0.49±0.02**	**0.19±0.05**
Galactinol	1±0.07	1.99±0.14	1.95±0.11	**20.11±3.53**	**19.16±2.68**	**18.9±1.96**
Raffinose	1±0.27	1.51±0.17	1.40±0.12	**38.59±8.24**	**50.21±6.07**	**45.6±3.40**
Glucose	1±0.09	1.56±0.20*	1.57±0.18*	**1.53±0.09**	1.18±0.05*	1.34±0.08*
myo-inositol	1±0.08	1.43±0.12*	1.45±0.11*	**3.97±0.20**	**3.84±0.35**	**4.78±0.13**
Xylose	1±0.11	1.08±0.03	1.00±0.05	**2.04±0.17**	**2.00±0.10**	**1.96±0.09**
Sucrose	1±0.16	1.26±0.12	1.24±0.03	**4.00±0.40**	**3.94±0.32**	**4.09±0.46**
Fucose	**1±0.05**	**0.98±0.04**	**1.01±0.12**	**1.34±0.8**	**1.14±0.05**	**1.06±0.09**
Fructose	**1±0.19**	**1.50±0.21**	**1.90±0.03***	**1.31±0.12**	**0.61±0.05***	**0.81±0.03***
Galactose /Mannose	**1±0.13**	**1.22±0.09**	**1.19±0.07**	**3.43±0.51**	**2.71±0.17**	**2.83±0.29**
Glycerol	**1±0.05**	**1.06±0.13**	**1.10±0.15**	**2.19±0.39**	**1.87±0.17**	**1.92±0.17**
Trehalose	1±0.06	1.13±0.13	1.09±0.11	1.52±0.29	**2.51±0.22***	**2.32±0.26***
4-OH-Proline	1±0.13	0.85±0.04	0.77±0.08	**6.10±0.52**	**5.58±0.33**	**5.68±0.51**
Spermidine	1±0.16	0.95±0.13	0.89±0.09	**2.06±0.13**	**2.27±0.10**	**2.16±0.09**
Putrescine	1±0.07	0.74±0.05	0.70±0.10	**3.20±0.45**	**3.53±0.38**	**3.30±0.46**
