# Supplementary material for: Decreased Levels of Thioredoxin o1 Influences Stomatal Development and Aperture but Not Photosynthesis under Non-Stress and Saline Conditions
Source: Int J Mol Sci. 2021 Jan 21;22(3):1063. doi: 10.3390/ijms22031063 (PMC7865980; doi:10.3390/ijms22031063)
Supplement: Supplementary file 1 [file ijms-22-01063-s001.zip › Supplementary Table S2.pdf]

***Supplementary Table 2.*** **Percentage (%) of increase (↑) or decrease (↓) of different parameters in two *Attrxo1* mutant lines (KO1 and KO2) related to wild type (WT) values of plants grown in the absence (Control) and presence (100 mM) of NaCl.** Results belong to similar parameters measured in the same lines but in different conditions: 150 µmol $m^{-2}$ $s^{-1}$ PAR, long photopheriod (16 h light/8 h dark) as described in Calderón et al. (2018) [29] and 80 µmol $m^{-2}$ $s^{-1}$ PAR, long photopheriod (16 h light/8 h dark) and grown in agar plates as reported in Sánchez-Guerrero et al. (2019) [10]. Only significant differences are presented when compared to WT in each condition (P<0.05) using the t-Student's test. ns: not significant.

| **CONTROL CONDITION** | **Present paper** | | **Calderón et al., 2018** | | **Sánchez-Guerrero et al., 2019** | |
|---|---|---|---|---|---|---|
| | **KO1** | **KO2** | **KO1** | **KO2** | **KO1** | **KO2** |
| Rosette diameter | ns | ns | ns | ns | ↓ 11.3 | ns |
| Leaf number | ↑ 17.0 | ns | ns | ns | ns | ↑ 7.6 |
| Fresh weight | ns | ns | ns | ns | ns | ↑ 59.0 |
| Total stomata/$mm^2$ | ns | ns | ↑ 47.7 | ↑ 53.5 | | |
| Stomatal index | ↑ 18.2 | ↑ 9.9 | ↓ 14.0 | ↓ 8.0 | | |
| $g_s$ | ↑ 27.3 | ↑ 20.3 | ns | ns | | |
| NO | ns | ns | ↑ 25.1 | ↑ 17.2 | | |
| $H_2O_2$ | ns | ns | ns | ns | | |
| Carbonyl proteins | ↓ 67.0 | ↓ 50.0 | ↑ 17.6 | ↑ 27.6 | | |
| MDA | ↑ 332 | ↑ 197 | ns | ns | | |
| ASC | ns | ns | ns | ns | | |
| GSH | ns | ns | ns | ns | | |
| GSSG | ns | ns | ns | ns | | |
| GSNO | ns | ↑ 21.0 | ns | ns | | |

| **100 mM NaCl** | **KO1** | **KO2** | **KO1** | **KO2** | **KO1** | **KO2** |
|---|---|---|---|---|---|---|
| Rosette diameter | ns | ns | ns | ns | ↓ 4.8 | ↓ 20.5 |
| Leaf number | ns | ns | ns | ns | ns | ↑ 5.7 |
| Fresh weight | ns | ns | ns | ns | ns | ns |
| Total stomata/$mm^2$ | ↑ 31.3 | ns | ↑ 14.4 | ns | | |
| Stomatal index | ns | ns | ↓ 6.7 | ↓ 11 | | |
| Stomatal conductance | ↑ 65 | ↑ 137 | ns | ns | | |
| NO | ↑ 48.3 | ↑ 33 | ↑ 8.3 | ns | | |
| $H_2O_2$ | ns | ns | ↑ 23.4 | ↑ 15.5 | | |
| Carbonyl proteins | ↓ 54.0 | ↓ 70.0 | ns | ns | | |
| MDA | ns | ns | ↑ 53.5 | ↑ 43.9 | | |
| ASC | ↑ 59.0 | ↑ 32.0 | ↑ 21.1 | ns | | |
| GSH | ns | ns | ↓ 19.0 | ns | | |
| GSSG | ns | ns | ↓ 21.0 | ns | | |
| GSNO | ns | ns | ns | ns | | |
