# Supplementary material for: Decreased Levels of Thioredoxin o1 Influences Stomatal Development and Aperture but Not Photosynthesis under Non-Stress and Saline Conditions
Source: Int J Mol Sci. 2021 Jan 21;22(3):1063. doi: 10.3390/ijms22031063 (PMC7865980; doi:10.3390/ijms22031063)
Supplement: Supplementary file 1 [file ijms-22-01063-s001.zip › FigureS1.pdf]

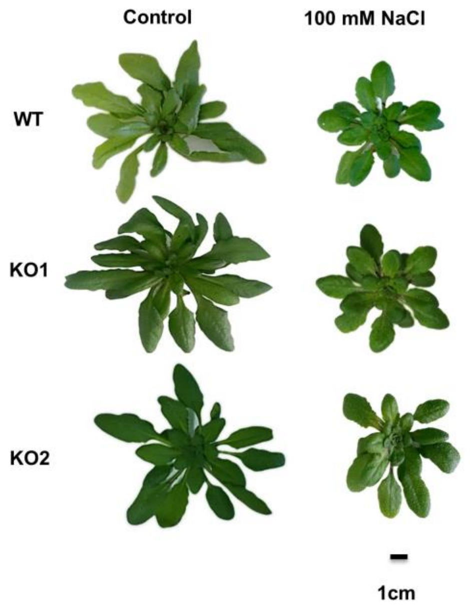

Figure S1. Arabidopsis plants of 33 days old representative of the genotypes WT and two KO *Attrxo1* lines grown in the absence (Control) and presence of 100 mM NaCl for 21 days.
